# PTEN and PIK3CA gene copy numbers and poor outcomes in non-small cell lung cancer patients with gefitinib therapy

**DOI:** 10.1038/bjc.2011.494

**Published:** 2011-11-17

**Authors:** M J Fidler, L E Morrison, S Basu, L Buckingham, K Walters, M Batus, K K Jacobson, S S Jewell, J Coon, P D Bonomi

**Affiliations:** 1 Section of Medical Oncology, Rush University Medical Center, 1725 West Harrison, Street 821, Chicago, IL 60612, USA; 2 Abbott Molecular Inc., 1300 East Toughy Avenue, Des Plaines, IL 60018-3315, USA; 3 Department of Preventative Medicine, Rush University Medical Center, 1700 West Van Buren Street, Room 470, Chicago, IL 60612, USA; 4 Department of Pathology, Rush University Medical Center, 1750 West Harrison Street, Chicago, IL 60612, USA

**Keywords:** EGFR, PTEN, PI3KCA, lung cancer, gefitinib

## Abstract

**Background:**

Preclinical studies in non-small cell lung cancer (NSCLC) suggest the interaction of PTEN and PI3K affects sensitivity to epidermal growth factor receptor (EGFR) tyrosine kinase inhibitors (TKIs). We investigated outcomes in relation to *PTEN*, *PIK3CA* and *EGFR* gene copy number, and chromosome 7 (CEN7) polysomy in NSCLC patients treated with gefitinib.

**Methods:**

Fluorescent *in situ* hybridisation analyses of *PTEN*, *PIK3CA*, *EGFR* and CEN7 were performed on tumour specimens from patients treated on the expanded access gefitinib trial. Progression-free survival (PFS) and overall survival (OS) were correlated with outcomes in all patients and *EGFR* wild-type patients.

**Results:**

Progression-free survival (hazard ratio=2.54, *P*<0.001) and OS (hazard ratio=4.04, *P*<0.001) were significantly shorter in patients whose tumours had all of the following molecular patterns: CEN7 <4 copies per cell, *PTEN* loss (<2 copies in at least 20% of cells), and *PIK3CA* gain (>2 copies in at least 40% of cells) both in all and *EGFR* wild-type only patients.

**Conclusion:**

The combination of low CEN7 copy number, *PTEN* loss, and *PI3KCA* gain may be useful for identifying NSCLC patients unlikely to benefit from treatment with EGFR (TKIs), specifically in wild-type *EGFR* cases.

Epidermal growth factor receptor (EGFR) tyrosine kinase inhibitors (TKIs) have provided a novel way to treat advanced non-small cell lung cancer (NSCLC; Shepherd *et al*, 2005; Thatcher Lancet *et al*, 2005). Non-small cell lung cancers containing activating *EGFR* mutations in exons 19 and 21 are associated with significantly higher response rates and superior progression-free survival (PFS) in patients treated with gefitinib compared with first-line platinum doublet regimens and to second-line single-agent docetaxel (Mok *et al*, 2009; Douillard *et al*, 2010; Maemondo *et al*, 2010; Mitsudomi *et al*, 2010). Though EGFR TKIs produce their most pronounced effects in patients with EGFR mutations, there is evidence that patients with wild-type EGFR also benefit from EGFR TKI treatment with significantly longer survival being observed with maintenance erlotinib in patients who had wild-type tumours (Cappuzzo *et al*, 2010). Similarly, there was a trend for longer survival in patients with wild-type tumours treated with erlotinib as second- or third-line treatment (Zhu *et al*, 2008).

The Identification of additional molecular markers predictive of clinical benefit with EGFR TKIs in wild-type tumours would have therapeutic and economic implications for NSCLC patients. In addition, molecular markers may identify rationale strategies for combining other targeted agents with EGFR TKIs.

PI3K and PTEN are key downstream components of the EGFR pathway and have significant roles in cell survival, proliferation, and growth (Cantley, 2002; Lou *et al*, 2003; Bianco *et al*, 2003a; Engelman, 2009). The expression of these enzymes has also been related to EGFR TKI resistance in preclinical models (Janmaat *et al*, 2003; Engelman *et al*, 2005; Yamasaki *et al*, 2007). Earlier work suggested an association between patient outcomes and the combination of high chromosome 7 copy number (CEN7; a surrogate for *EGFR* copy number gain due to polysomy) and PTEN expression in advanced NSCLC patients treated with gefitinib (Buckingham *et al*, 2007). The objective of this study is to evaluate potential relationships between gene copy numbers for *PIK3CA* (the catalytic subunit of PI3K), *PTEN* and *EGFR*, and outcomes for NSCLC patients treated with gefitinib.

## Materials and methods

This was a retrospective analysis of specimens from 82 patients in the gefitinib Expanded Access Trial, treated for >1 week, and collected as described previously. (Buckingham *et al*, 2007) Eligibility criteria and methods for establishing clinical data were also described. Chart review and this study's analyses were approved by the Rush University Medical Center Institutional Review Board. In general, patients were previously treated with at least one chemotherapy regimen or were considered ineligible for chemotherapy.

Based on previous work showing higher survival with PTEN expression by immunohistochemistry (IHC), an exploratory analysis was undertaken to study the influence of PTEN copy number loss and PIK3CA copy number gains in conjunction with EGFR alterations by fluorescent *in situ* hybridisation (FISH) on the same gefitinib cohort previously analysed. As no prospectively validated EGFR FISH analysis has been published to date, EGFR gene copy number status was assessed using multiple measures: the average number of *EGFR* copies per cell (EGFR/cell), the average number of chromosome 7 copies per cell (CEN7/cell), the average number of *EGFR* copies per chromosome 7 copies (EGFR/CEN7), the percentage of cells with more copies of *EGFR* than chromosome 7 (EGFR/CEP7 gain), the percentage of cells with >2 copies of *EGFR* (EGFR gain), and the percentage of cells with >2 copies of chromosome 7 (CEN7 gain). Fluorescence *in situ* hybridisation analyses were carried out as follows. The formalin-fixed paraffin-embedded lung tumour tissues and cell pellets were analysed with a dual colour probe set (Abbott Molecular Inc, Desplains, IL, USA) comprising SpectrumOrange LSI EGFR and SpectrumGreen CEP 7 and a four-colour probe set comprising a probe spanning *PTEN* (LSI PTEN) labelled with SpectrumRed, a probe spanning *PIK3CA* labelled with SpectrumGold, and probes containing peri-centromeric repeat sequences specific for chromosomes 3 (CEN 3) and 10 (CEN 10) (SpectrumGreen CEP 3 and SpectrumAqua CEP 10). Probes with CEP and LSI designations were obtained from Abbott Molecular Inc., and the four-colour probe set has been previously described. (Morrison *et al*, 2007) Fluorescence *in situ* hybridisation signals were enumerated in ⩾40 cells per specimen to obtain copy numbers for each locus, and 72 specimens yielded results for all six probes. Gene copy number gain for *EGFR*, *PTEN*, and *PIK3CA* was defined as >2 gene copies per cell. Conversely, gene loss was defined as <2 copies per cell. Epidermal growth factor receptor/CEN7 was deemed high if the ratio was >1. The response variables considered include, PFS and overall survival (OS). The status of EGFR mutation (exons 19 and 21) was obtained for 55 of the specimens (as described in Buckingham *et al*, 2007). Statistical analyses were carried out on the total population of patients with FISH analyses and repeated for the EGFR wild-type and mutant populations. Descriptive statistics were obtained and Fisher's exact test was used to measure the association among recurrence, survival, and categorised covariates. For purposes of tabular and time-to-even analyses, the biomarker measurements were divided into two classes (high/low) using optimally chosen marker specific thresholds in the absence of prior published cutoffs for *PTEN* and *PI3KCA* FISH testing. The differences in OS and PFS between the low and high biomarker groups were assessed by the log-rank test and Kaplan–Meier method was used to obtain estimates of OS and PFS curves. Predictors that were statistically significant or marginally significant in univariate analyses or were deemed to be clinically or biologically important were included as candidate covariates in multivariate Cox proportional hazards (PHs) regression models. Statistical analyses were performed using Version 9.2 of the SAS software, Version 7.0 of the JMP software (SAS Institute, Cary, NC, USA) and the statistical software R. All reported *P*-values are two sided and *P*-values between (0.05–0.10), (0.01–0.05), and (<0.01) are reported as marginally significant, significant, and strongly significant, respectively.

## Results

The majority of patients included in this study were over the age of 60 (77%). In all, 54% were female, 85% had a smoking history, 69% had adenocarcinoma, 57% had Eastern Oncology Cooperative Group performance status of 0–1, and 83% had received previous chemotherapy as detailed in Table 1. The gain of PIK3CA is marked as >2 *PIK3CA* copies and high PIK3CA gain is measured as the percentage of cells with PIK3CA gain being ⩾40%. Similarly, a cutoff of 20% is used for PTEN loss (percentage of cells with <2 *PTEN* copies). The associations of PIK3CA gain, PTEN loss and the six EGFR-related parameters with PFS and OS were assessed by univariate analyses in Table 2. Chromosome 3 and CEN10 copy numbers did not provide useful associations with outcomes and were not included in the table. Of the six *EGFR*-related parameters examined, only CEN7/cell, cutoff=4.0, provided statistically significant classification with respect to OS (*P*=0.02). Epidermal growth factor receptor/CEN7 gain, cutoff=34% (*P*=0.02), and EGFR/CEN7, cutoff >1.0 (*P*=0.04), were the only *EGFR*-related parameters to provide statistically significant classification with respect to PFS. Individually, *PIK3CA* did not show a statistically significant relationship with the above endpoints, whereas high PTEN loss did correlate with worse OS (*P*=0.01).

**Figure 1:**
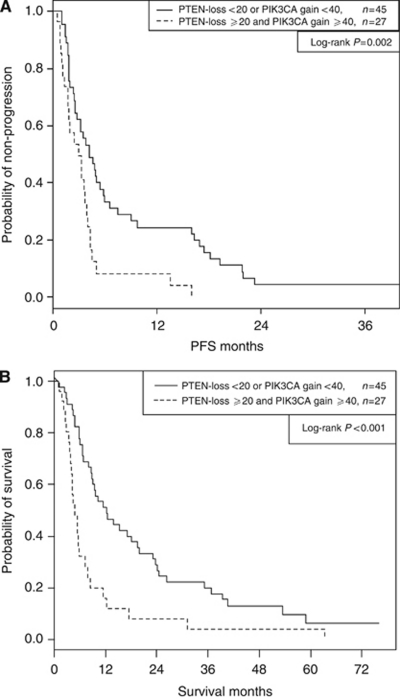
Progression free survival (PFS) and OS by PTEN loss and PIK3CA gain. (**A**) Progression free survival in all patients. (**B**) Overall survival in all patients.

**Figure 2:**
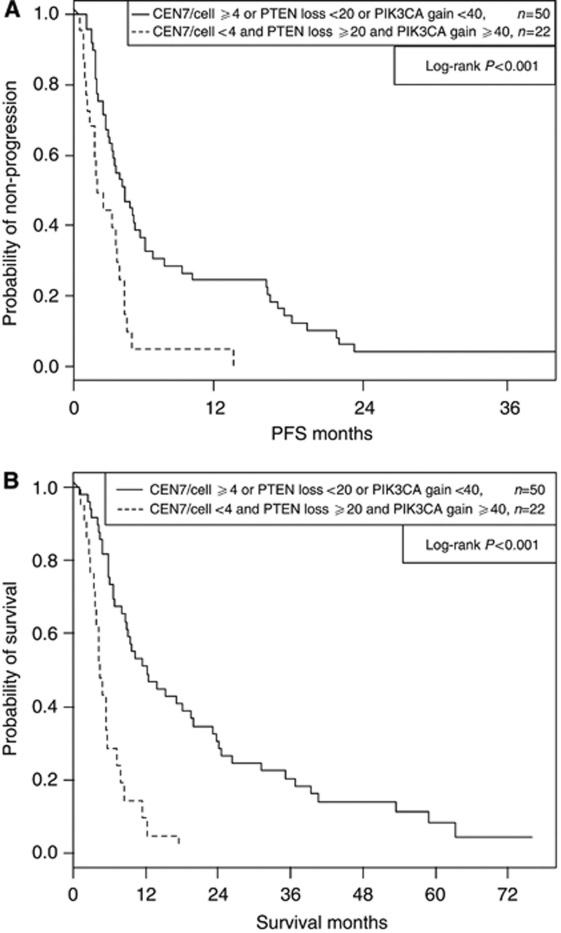
Progression-free survival(PFS) and OS by PTEN loss, PIK3CA gain and chromosome 7 (CEN7) polysomy. (**A**) Progression free survival in all patients. (**B**) Overall survival in all patients.

Thirty-eight percent of 72 patients had the double combination of high levels of PIK3CA gain (cutoff=40%) and high levels of PTEN loss (cutoff=20%) and had strongly significantly shorter PFS (*P*=0.002) and OS (*P*<0.001) than the remaining patients (3.02 *vs* 4.27 months median PFS, 4.93 *vs* 12.3 months median OS). Corresponding PFS and OS curve estimates are plotted in (Figure 1 A and B), respectively. Thirty-one percent of the 72 patients had the triple combination of low CEN7/cell (cutoff=4), high PIK3CA gain (cutoff=40%) and high PTEN loss (cutoff=20%), and experienced further shortened PFS (*P*<0.001) and OS (*P*<0.001) than the remaining patients (2.04 *vs* 4.21 months median PFS, 4.34 *vs* 12.3 months median OS). Corresponding PFS and OS curve estimates are plotted in (Figure 2A and B), respectively, for this three variate combination. Several *EGFR*-related parameters other than CEN7/cell provided highly significant associations in combination with PTEN loss and PIK3CA gain. Low EGFR gain (cutoff=75%), high PTEN loss, and high PIK3CA gain provided highly significant associations with PFS (*P*<0.001) and OS (*P*<0.001), and grouping low EGFR/CEN7 gain (cutoff=34%) with the same PTEN and PIK3CA parameters also provided high association with PFS (*P*=0.002) and OS (*P*<0.001). See Table 3 for double and triple covariate analyses.

**Figure 3:**
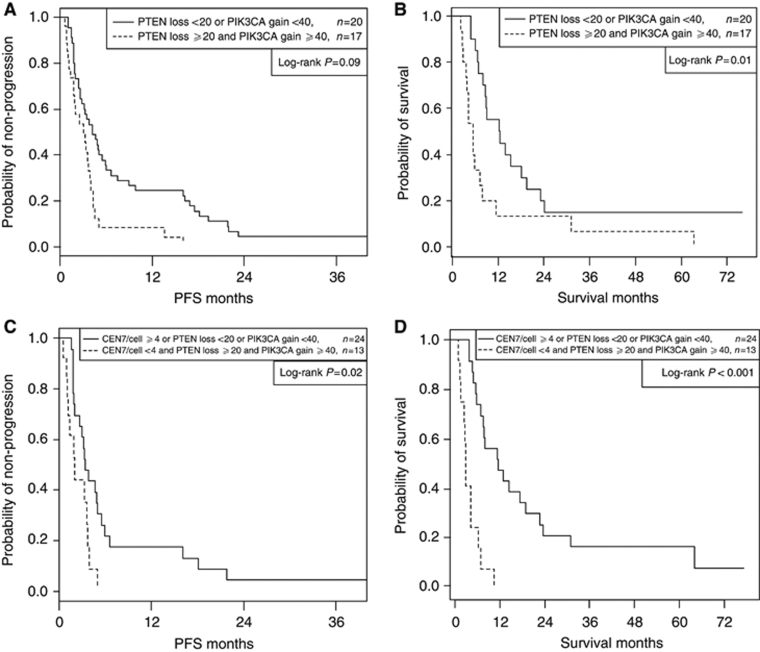
(**A**) Progression-free survival in EGFR wild-type patients (two covariates). (**B**) Overall survival in EGFR wild-type patients (two covariates). (**C**) Progression-free survival in EGFR wild-type patients (three covariates). (**D**) Overall survival in EGFR wild-type patients (three covariates).

Seventeen of 55 patients tested had an *EGFR* activating gene mutation in exon 19 or exon 21. Patients with *EGFR* gene mutation had superior PFS compared with *EGFR* wild-type patients (13.6 *vs* 3.25 months median PFS, *P*=.003) but the association with OS was not significant (23.8 *vs* 7.98 months OS, *P*=0.18). In the 37 patients with wild-type *EGFR* and complete FISH data, patients with both high PIK3CA gain (cutoff=40%) and high PTEN loss (cutoff=20%) showed marginally significant shortening of PFS (3.02 *vs* 3.55 months median PFS, *P*=0.09) and significant shortening of OS (5.49 *vs* 12.3 months OS, *P*=0.01). Corresponding PFS and OS curve estimates are plotted in (Figure 3A and B), respectively. Wild-type patients with the triple combination of low CEN7/cell (cutoff=4), high PIK3CA gain (cutoff=40%), and high PTEN loss (cutoff=20%) showed significant shortening of PFS (2.04 *vs* 3.35 months, *P*=0.02) and strongly significant shortening of OS (4.18 *vs* 12.5 months, *P*<0.001). Corresponding PFS and OS curve estimates for patients with and without this triple combination are plotted in Figure 3C and D, respectively. Triple combinations using EGFR/CEN7 gain and EGFR gain were also significant and are presented in Table 3. Of note, ∼30% of wild-type patients presented with an unfavourable triple combination and had shortened PFS and OS. In the small group of 15 patients with activating *EGFR* mutations and complete FISH data, the combination of low CEN7/cell (cutoff=4), high PIK3CA gain (cutoff=40%), and high PTEN loss (cutoff=20%) showed marginally significant shortening of OS (*P*=0.07).

The results of multivariate Cox PHs regression analysis are listed in Table 4 for the subset of patients tested for EGFR mutations with full FISH data (*N*=52). Chromosome 7/cell (cutoff=4), PTEN loss (cutoff=20%) and PIK3CA gain (cutoff=40%) continued to have strongly significant association with OS after adjusting for gender, smoking status and histology, and PIK3CA gain was marginally associated with PFS. Epidermal growth factor receptor mutation did not show a significant association with OS, but had strongly significant association with PFS.

## Discussion

Patients with *EGFR* mutations in exons 19 and 21 have been shown to have significantly higher response rates and improved PFS when treated with frontline gefitinib, and *EGFR* mutation has become an established criterion for selecting an EGFR TKI as first-line therapy in stage IV NSCLC patients. (Mok *et al*, 2009; Maemondo *et al*, 2010; Mitsudomi *et al*, 2010). In the absence of an *EGFR*-activating gene mutation, it seems likely that a functional EGFR pathway is necessary for EGFR TKIs to be effective. Much work has been done that correlates retrospectively applied *EGFR* gene copy number with outcomes in NSCLC patients treated with EGFR TKIs (Cappuzzo *et al*, 2005; Tsao *et al*, 2005; Zhu *et al*, 2008). A recent meta-analysis showed that high *EGFR* gene copy number was associated with longer survival in NSCLC patients treated with an EGFR TKI (Dahebreh *et al*, 2011). The most commonly applied criteria for FISH positivity is complex, has yet to be validated when prospectively applied to a clinical trial and seems to have less importance when comparing outcomes in patients treated with TKIs *vs* second-line chemotherapy (Douillard *et al*, 2010). In this study, an exploratory analysis was conducted measuring EGFR copy number in several different ways. Both high chromosome 7 copy number and a high ratio of *EGFR* to chromosome 7 copy numbers were associated with either prolonged OS or PFS.

Preclinical work suggests that the striking benefit of EGFR TKI therapies in mutation-positive tumours is related to massive apoptosis (Sordella *et al*, 2004). involving PI3K and PTEN. Recently, PTEN loss has been suggested as a potential mechanism of EGFR TKI resistance in NSCLC, which contain activating EGFR mutations (Sos *et al*, 2009). In addition, multivariate analysis in our initial studies showed that PTEN expression, detected by IHC, was significantly related to OS in gefitinib-treated patients (Buckingham *et al*, 2007). These considerations suggested PTEN might also be a determinant of the efficacy of EGFR TKIs in EGFR wild-type tumours and prompted us to evaluate *PTEN* and *PIK3CA* gene copy number in our gefitinib-treated patients. Although *PTEN* gene copy number alone was significantly related to OS, this was not the case for *PIK3CA*. However, the combination of gene copy data for *PTEN* and *PIK3CA* was strongly associated with both PFS and OS, and may be a more useful stratification than PTEN alone (Table 3).

The most powerful correlate of improved survival was the combination of *EGFR* or chromosome 7 copy number with *PIK3CA* and *PTEN* copy numbers. Although our results were obtained in a relatively small, single arm study, gefitinib-treated patients whose tumours contained low CEN7/cell (or low EGFR/CEN7 gain or low EGFR gain), high PTEN loss, and high PIK3CA gain had significantly shorter PFS and OS than other patients. The poor outcome with this molecular signature was seen both in the entire group and in the wild-type EGFR subset. The subset of patients with EGFR mutations and complete FISH data (*n*=15) was too small to support strong conclusions in this study. We are currently evaluating this set of markers in a larger group of patients treated with erlotinib. If our ongoing study shows similar results, this molecular profile, which was found in 30% of our patients, may identify a significant subset of NSCLC patients who derive minimal or no benefit from treatment with EGFR TKI.

In addition to the clinical implications of excluding patients who are unlikely to benefit from treatment with EGFR inhibitors, this selection strategy might have significant economic impact. Bradbury *et al* (2010) recently reported that the cost–benefit ratio for erlotinib was marginal. Subset analysis showed that the cost–benefit ratio was more favourable in never smokers and in patients with high *EGFR* gene copy numbers. The authors recommended increasing efforts to identify the most cost-effective way to use EGFR TKIs.

Our observations might also be useful in designing combination regimens targeting both the EGFR TKIs and downstream pathways. Preclinical studies have shown that reduced PTEN expression increases cancer cell survival and proliferation, and has been associated with resistance to EGFR inhibitors in NSCLC and colon cancers, and resistance to trastuzumab in breast cancer (Bianco *et al*, 2003b; Fujita *et al*, 2006; Berns *et al*, 2007; Sierra *et al*, 2010). It was somewhat surprising to find that a relatively high percentage of tumours (80%) had PTEN gene loss (defined as ⩾20% of cells with <2 copies of PTEN), and that relatively subtle PTEN loss was associated with significantly shorter PFS and OS. If additional studies yield similar results, relatively minor alterations in wild-type *PTEN* gene copy number might have prognostic and therapeutic implications for NSCLC patients in identifying a practical patient group likely to benefit from multi-targeted therapy.

Our observation that the combination of *PTEN* and *PIK3CA* gene copy number more strongly related to outcome than either marker alone is consistent with recent results reported in breast cancer patients treated with trastuzumab Berns *et al* (2007). observed higher rates of disease progression on trastuzumab in breast cancer patients with either *PIK3CA* mutations or with low PTEN expression. Further, they suggested that assessing both molecular markers might be required for optimal prediction of disease progression during the treatment with trastuzumab. These preliminary observations might be particularly pertinent xin defining the roles of PI3K inhibitors and mTOR inhibitors in NSCLC.

In summary, if our results with CEN7, *EGFR*, *PIK3CA*, and *PTEN* FISH analyses are confirmed in larger groups of patients, this molecular profile could have clinical and economic implications for patients being considered for EGFR TKI treatment. Similarly, if our ongoing *PTEN* and *PIK3CA* gene copy number study in erlotinib-treated patients shows results consistent with our observations in gefitinib-treated patients, evaluation of *PTEN* and *PIK3CA* gene copy numbers should be considered in single agent and combination trials testing PI3K and mTOR inhibitors in NSCLC patients.

## Figures and Tables

**Table 1:** Patient characteristics

**Characteristic**	**Number of patients (%)**	**Objective response (%)**
Total	82 (100)	12 (15)
		
*Age (years)*		
⩾60	62 (77)	8 (13)
<60	20 (23)	4 (20)
		
*Gender*		
Male	37 (46)	5 (14)
Female	44 (54)	7 (16)
		
*Smoking status*		
Yes	70 (85)	5 (7)
Never smoked	12 (15)	7 (58)
		
*Histopathological subtype*		
Adenocarcinoma	56 (69)	10 (18)
Other	26 (32)	2 (8)
		
*Performance status*		
(0–1)	46 (57)	6 (13)
(2–4)	34 (43)	6 (17)
		
*Previous chemotherapy*		
None	14 (17)	2 (14)
One	39 (49)	7 (18)
Two or more	28 (34)	3 (11)

**Table 2:** Univariate analyses

**Biomarkers**		**Patients (%)**	**Median PFS months**	**Hazard ratio**	***P*-value (log-rank test)**	**Median survival months**	**Hazard ratio**	***P*-value (log-rank test)**
*EGFR/cell*								
Low	<4.5	59 (73)	2.53			7.27		
High	⩾4.5	22 (27)	4.31	0.78	0.32	9.44	0.74	0.24
								
*CEN7/cell*								
Low	<4	67 (83)	2.86			6.9		
High	⩾4	14 (17)	4.04	0.78	0.42	17.2	0.49	0.02
								
*EGFR/CEN7*								
Low	⩽1	24 (27)	2.04			5.95		
High	>1	57 (73)	4.04	0.54	0.02	8,78	0.66	0.11
								
*EGFR/CEN7 gain*								
Low	<34%	41 (51)	2.47			6.61		
High	⩾34%	40 (49)	4.32	0.58	0.02	11.2	0.68	0.1
								
*EGFR- gain*								
Low	<70%	52 (64)	2.53			7.27		
High	⩾70%	29 (36)	4.31	0.82	0.43	11.5	0.73	0.2
								
*CEN7 gain*								
Low	<80%	68 (84)	3.22			7.27		
high	⩾80%	13 (16)	4.32	0.73	0.31	17.2	0.62	0.12
								
*PTEN loss*								
Low	<20%	15 (21)	5.92			20		
High	⩾20%	58 (79)	3.25	1.47	0.19	6.9	2.13	0.01
								
*PI3KCA gain*								
Low	<40%	33 (46)	3.52			9.6		
High	⩾40%	39 (54)	3.61	1.46	0.13	6.64	1.31	0.27

Abbreviations: CEN7=chromosome 7; EGFR=epidermal growth factor receptor; PFS=progression-free survival; PI3KCA=phosphatidylinositol 3-kinase catalytic subunit alpha; PTEN=phosphatase and tensin homologue.

**Table 3:** Two and three markers analyses

**Biomarkers**	**Patients**	**%**	**Median PFS months**	**Hazard ratio**	***P*-value (log-rank test)**	**Median survival months**	**Hazard ratio**	***P*-value (log-rank test)**
*High PTEN loss and high PIK3CA gain*								
No	45	62	4.27			12.3		
Yes	27	38	3.02	2.23	0.002	4.93	2.38	<0.001
								
*Low CEN7/cell, high PTEN loss, and high PIK3CA gain*								
No	50	69	4.21			12.26		
Yes	22	31	2.04	2.54	<0.001	4.34	4.04	<0.001
								
*Low EGFR/CEN7 gain, high PTEN loss, and high PIK3CA gain*								
No	56	78	4.04			10.4		
Yes	16	22	2.04	2.62	0.002	4.93	3.37	<0.001
								
*Low EGFR gain, high PTEN loss, and high PIK3CA gain*								
No	55	76	4.04			10.36		
Yes	17	24	2.04	2.52	0.003	4.93	3.35	<0.001
								
Wild Type (n=37)								
*High PTEN loss and high PIK3CA gain*								
No	20	54	3.55			12.3		
Yes	17	46	3.02	1.84	0.09	5.49	2.41	0.012
								
*Low CEN7/cell, high PTEN loss, and high PIK3CA gain*								
No	24	65	3.35			12.5		
Yes	13	35	2.04	2.41	0.02	4.18	6.77	<0.001
								
*Low EGFR/CEN7 gain, high PTEN loss, and high PIK3CA gain*								
No	27	73	3.35			10.6		
Yes	10	27	2.04	1.94	0.098	5.49	3.97	0.001
								
*Low EGFR gain, high PTEN loss, and high PIK3CA gain*								
No	26	70	3.25			10.64		
Yes	11	30	2.04	1.84	0.13	5.49	3.97	0.001
EGFR Mutant (n=15)								
*High PTEN loss, high PIK3CA gain yes/no*								
No	12	80	16.31			26.4		
Yes	3	20	4.54	5.01	0.02^*^	5.57	8.14	0.002
								
*Low CEN7/cell, high PTEN loss, and high PIK3CA gain*								
No	13	87	16.31			26.4		
Yes	2	13	9.07	3.07	0.2^*^	11.5	4.35^*^	0.07

Abbreviations: CEN7=chromosome 7; EGFR=epidermal growth factor receptor; PFS=progression-free survival; PI3KCA=phosphatidylinositol 3-kinase catalytic subunit alpha; PTEN=phosphatase and tensin homologue.

^*^P-values, though statistically significant, are reflective of a small sample size.

**Table 4:** Multivariate Cox proportional hazards regression of patients with EGFR mutation status

	**PFS HR**	***P*-value**	**OS**	***P*-value**
Female	1.19	0.6	0.77	0.45
Smoker	1.48	0.35	1.32	0.53
Adenocarcinoma	1.49	0.35	1.06	0.9
EGFR mutation	0.32	0.006	0.74	0.47
CEN7/cell <4	0.99	0.97	3.36	0.006
PTEN loss ⩾20%	1.62	0.26	4.06	0.002
PIK3CA gain ⩾40%	2	0.06^*^	3.02	0.003
*n*=52				

Abbreviations: CEN7=chromosome 7; EGFR=epidermal growth factor receptor; HR=hazard ratio; PFS=progression-free survival; PI3KCA=phosphatidylinositol 3-kinase catalytic subunit alpha; PTEN=phosphatase and tensin homologue.
